# Supplementary material for: Punicalagin Prevents Inflammation in LPS- Induced RAW264.7 Macrophages by Inhibiting FoxO3a/Autophagy Signaling Pathway
Source: Nutrients. 2019 Nov 15;11(11):2794. doi: 10.3390/nu11112794 (PMC6893462; doi:10.3390/nu11112794)
Supplement: Supplementary file 1 [file nutrients-11-02794-s001.pdf]

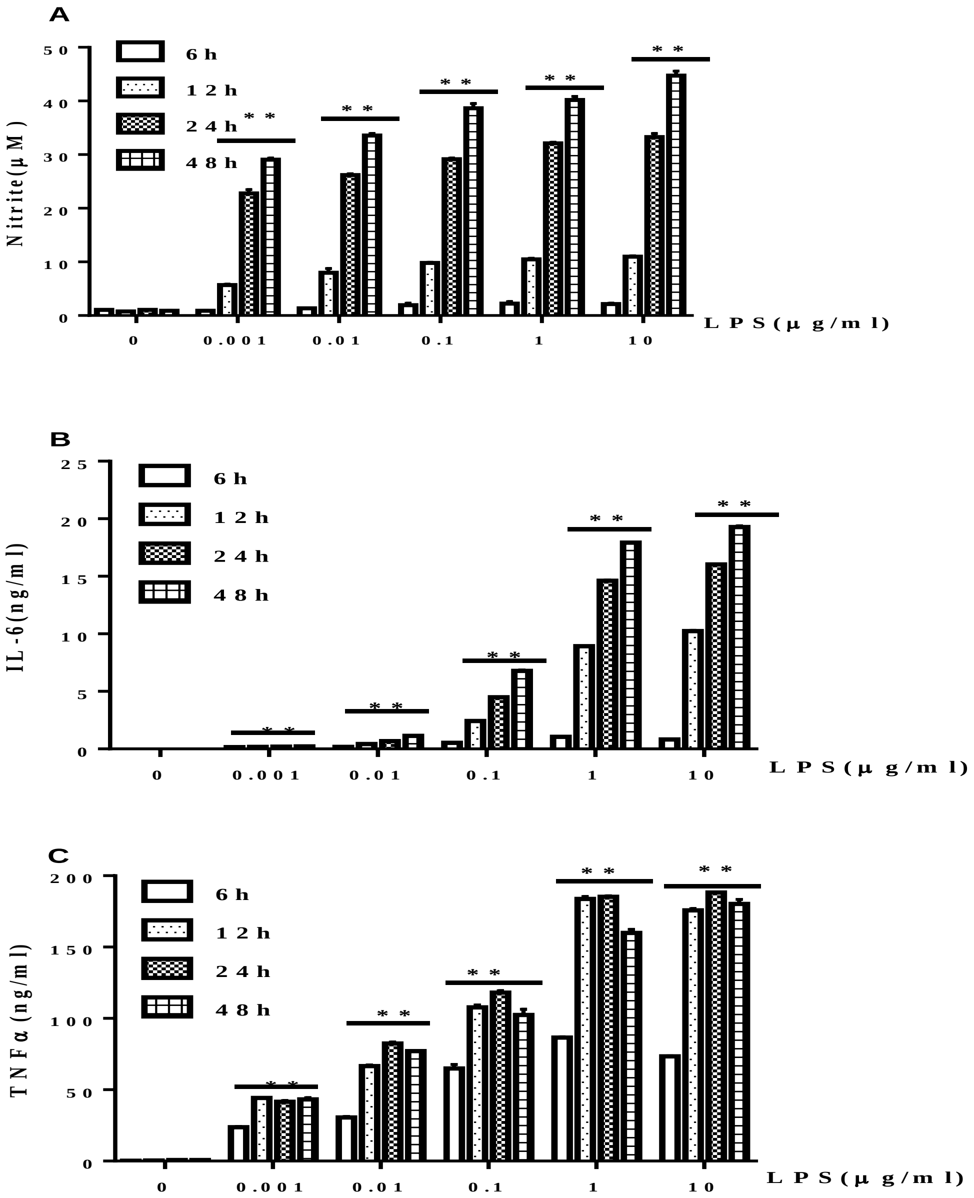

Figure S1. LPS-induced RAW264.7 macrophages inflammatory responses. (**A-C**) Cells were pretreated with various concentrations of LPS for 6, 12, 24, 48 h. The NO content was determined by Griess reagent and the production of cytokines were measured by Enzyme-linked immunosorbent assay (ELISA) kit using the microplate reader. The data are presented as means ± SD(n = 3). (*P < 0.05, **P < 0.01 vs Control group)
